# Ethical Decision-Making in Entrepreneurial Dentistry: An Exploratory Conceptual Framework Informed by Romanian Healthcare Studies

**DOI:** 10.3390/dj14090537

**Published:** 2026-08-27

**Authors:** George Dumitru Constantin, Alexandru Cucui-Cozma, Ioana Veja, Crisanta-Alina Mazilescu, Roxana Oancea

**Affiliations:** 1 Discipline of Clinical Skills, Department I Nursing, “Victor Babeș” University of Medicine and Pharmacy Timișoara, 300041 Timișoara, Romania; george.constantin@umft.ro; 2 Doctoral School, “Victor Babeș” University of Medicine and Pharmacy Timișoara, Eftimie Murgu Square 2, 300041 Timișoara, Romania; 3 Center for Advanced Research in Cardiovascular Pathology and Hemostaseology, “Victor Babeș” University of Medicine and Pharmacy Timișoara, 300041 Timișoara, Romania; 4 Department of Surgery II, Discipline of Surgery I, “Victor Babeș” University of Medicine and Pharmacy Timișoara, 300041 Timișoara, Romania; cucui.alexandru@umft.ro; 5 Department of Dental Medicine, Faculty of Dentistry, “Vasile Goldiș” Western University of Arad, 310025 Arad, Romania; 6 Teacher Training Department, Politehnica University Timișoara, 300596 Timișoara, Romania; 7 Translational and Experimental Clinical Research Centre in Oral Health, Department of Preventive, Community Dentistry and Oral Health, Faculty of Dental Medicine, “Victor Babeș” University of Medicine and Pharmacy Timișoara, 300041 Timișoara, Romania; roancea@umft.ro

**Keywords:** dental ethics, dental education, moral reasoning, ethical decision-making, private dentistry, DIT, ATBEQ, Idealism/Legalism, conditional responding

## Abstract

**Background/Objectives:** Dental graduates make clinical decisions shaped by ethical principles, legal obligations, patient expectations, and private-practice economics. This study synthesized empirical findings from Romanian healthcare education and practice and proposes an exploratory model for teaching ethical decision-making in entrepreneurial dentistry. **Methods:** Exploratory conceptual framework development informed by four previously published Romanian healthcare studies examining business-ethics attitudes using the Attitudes Toward Business Ethics Questionnaire (ATBEQ), moral reasoning using an adapted Defining Issues Test-2 (DIT-2; 242 valid cases), scenario-based ethical decision-making (n = 244), and ethical reasoning profiles derived from the joint administration of the ATBEQ and 35-item scenario questionnaire (n = 277). **Results:** “It depends” accounted for 47.8% of scenario responses. Students and practitioners differed regarding vaccination of healthcare workers, risk-based insurance contributions, and organ-market policy (chi-square, df = 2, uncorrected). Practitioners endorsed law-oriented arguments more strongly but weighted patient autonomy more heavily at end of life. Idealism/Legalism showed an isolated exploratory association with fewer conditional responses (B = −0.69, 95% CI −1.28 to −0.11, *p* = 0.021); however, the overall regression model was not statistically significant and had low explanatory power (R^2^ = 0.04, adjusted R^2^ = 0.02, *p* = 0.08). Four ethical reasoning profiles were identified. **Conclusions:** The G.D.C. framework is a theory-informed, hypothesis-generating educational framework derived from mixed Romanian healthcare samples. It has not been validated specifically in entrepreneurial dentistry and requires prospective, dental-specific validation.

## 1. Introduction

Dental education has traditionally emphasized biomedical competence, clinical skill, patient safety, and adherence to professional ethical principles. These remain essential foundations of dental practice. The classical principles of biomedical ethics, namely beneficence, non-maleficence, autonomy, and justice, continue to provide a basic normative framework for healthcare decision-making [1]. However, contemporary dental practice also requires graduates to make decisions in environments shaped by private-practice sustainability, treatment costs, technological investment, patient demand, competition, professional regulation, and legal accountability.

This creates a specific educational challenge. Students are often introduced to ethics through general principles and simplified dilemmas, whereas practitioners must apply ethical reasoning in situations in which several treatment options may be clinically acceptable but differ in cost, prognosis, esthetic value, accessibility, and long-term maintenance. In such cases, ethical decision-making cannot be reduced to abstract knowledge of principles. It requires the ability to integrate clinical evidence, patient autonomy, affordability, informed consent, legal boundaries, and the financial sustainability of practice.

Ethical decision-making in dentistry should also consider social vulnerability, disability, and limited health literacy, as these factors may affect access to care, treatment understanding, affordability, and participation in informed decision-making [2].

This tension is especially visible in private and mixed public–private dental systems. Economic rationality is not inherently incompatible with ethical dentistry. Dental practices require financial stability, trained staff, high-quality materials, adequate equipment, and organizational continuity. The ethical problem emerges when economic reasoning becomes insufficiently transparent, when treatment communication is selectively framed, or when the patient’s welfare is subordinated to commercial incentives.

Previous empirical work in Romanian healthcare education and practice provides several relevant findings. Attitudes Toward Business Ethics Questionnaire (ATBEQ)-based research showed that Romanian medical students and doctors tend to rely predominantly on moral objectivism in business-ethics reasoning, suggesting a preference for rational decisions grounded in objective moral standards [3]. Scenario-based ethical research showed that “It depends” was the most frequent response pattern across ethical dilemmas, indicating that participants often approached moral decisions conditionally rather than categorically [4]. Research based on an adapted Defining Issues Test-2 (DIT-2) showed that physicians more strongly endorsed law-based and conventional reasoning than students, while students showed greater variability and openness to compassion-driven justifications [5]. Finally, a profile-based study in which the ATBEQ and a 35-item clinical-dilemma questionnaire were administered jointly to 277 participants identified four ethical reasoning profiles and found that abstract moral orientations explained only a small share of conditional ethical responding [6].

Taken together, these findings suggest that ethical reasoning in healthcare and dental contexts may be better understood as a hybrid process than as a simple transition from idealism to pragmatism. The present study therefore proposes the G.D.C. model, or Grid for Decision-making in entrepreneurial Contexts, as an exploratory educational framework for dental ethics education. The model integrates three interacting filters: individual ethical orientation, entrepreneurial contextual pressure, and the normative-deontological safety framework. It also incorporates shared decision-making, ethical justification, communication, documentation, and reassessment as an iterative process that allows preliminary recommendations to be revised.

The primary focus of this study is educational: to propose a framework that helps dental students and practitioners develop structured ethical decision-making skills. Patient diversity and vulnerability are considered only as contextual factors that may influence ethical reasoning and communication.

The objectives of this conceptual study were: (1) to integrate previously published findings on moral reasoning, business-ethics attitudes, and scenario-based ethical decision-making among Romanian healthcare students and practitioners [3,4,5,6]; (2) to examine conditional ethical responses as an observed form of conditional responding, without assuming that they represent contextual ethical competence; (3) to reinterpret the previously published ethical reasoning profiles in a dental-education context [6]; and (4) to propose the G.D.C. model as an exploratory framework for teaching ethics in entrepreneurial dental practice.

The novelty of this study lies in integrating previously findings into the G.D.C. framework, a structured conceptual approach for dental ethics education. This framework may support more systematic analysis of clinical, patient-related, economic, and regulatory factors in complex treatment decisions. The present study does not replicate the primary analyses of the four previous studies. Instead, it integrates their findings on moral reasoning, business-ethics attitudes, conditional ethical responding, and ethical reasoning profiles [3,4,5,6] into a dentistry-oriented educational framework. Its main contribution is the development of the G.D.C. model as an exploratory pedagogical tool for case-based ethics education in entrepreneurial dental practice.

## 2. Materials and Methods

### 2.1. Study Design

This study was designed as an exploratory conceptual framework paper informed by four previously published Romanian healthcare studies [3,4,5,6]. The studies were purposively used because they provide complementary findings on business-ethics attitudes, moral reasoning, scenario-based conditional responding, and ethical reasoning profiles. They were not identified through a systematic literature search and were not statistically pooled or reanalyzed.

The purpose of the present study is not to conduct an integrative review or a new secondary-data analysis, but to conceptually integrate these previously published findings with relevant ethical theory in order to develop the G.D.C. framework for dental ethics education. All quantitative results presented in this study derive from the original source studies and should not be interpreted as new empirical findings.

The study was non-interventional. No clinical procedures were performed, no patient treatment was modified, and no patient-identifiable clinical data were collected.

### 2.2. Relationship with Previous Empirical Studies

The empirical findings synthesized in this study derive from four related studies previously conducted by the authors [3,4,5,6]. The present study provides a conceptual reinterpretation of those findings; it reports no new primary data and no new statistical analyses, and all quantitative results presented here are drawn from the cited source studies. Its added contribution lies in translating these findings into the G.D.C. model, a pedagogical tool for dental ethics training in entrepreneurial practice contexts.

The quantitative findings presented in this study were previously reported in the four source studies [3,4,5,6] and are not presented as new empirical evidence. The original contribution of the present work lies in their conceptual integration and reinterpretation within a dentistry-oriented educational framework. Specifically, the G.D.C. model organizes these findings into three interacting dimensions relevant to ethical decision-making in entrepreneurial dental practice and translates them into potential educational applications.

### 2.3. Empirical Sources and Analytical Samples

Four previously published empirical sources informed the conceptual framework.

First, business-ethics attitudes were examined using the ATBEQ in a sample that included 53 medical students, 192 doctors, and 108 management students [3]. The ATBEQ component was used to identify attitudes toward Machiavellianism, moral objectivism, social Darwinism, ethical relativism, and legalism. For the purposes of the present dental education model, the most relevant groups were medical students and doctors, because they provide insight into how healthcare training and professional practice relate to business-ethics reasoning.

Second, moral reasoning based on an adapted DIT was examined in a cohort of 244 respondents, including 51 senior medical students and 193 practicing physicians; after data cleaning, 242 valid cases were retained for analysis [5]. Within the student cohort, 82.35% were enrolled in dentistry and 17.65% in general medicine. Within the physician cohort, 68.39% were dentists and 31.61% were general practitioners. The physician group had 1–28 years of professional experience, with a median of 12 years.

Third, scenario-based ethical decision-making was examined in the same cohort of 244 participants [4]. The questionnaire included 35 items across seven ethical domains.

Fourth, the ethical profile component derives from a separate cross-sectional study in which the ATBEQ and the 35-item clinical-dilemma questionnaire were administered jointly to the same 277 participants [6]. This sample was recruited online through convenience sampling and comprised predominantly women (77.6%) and practicing physicians (67.9%), with medical and dental students accounting for 19.5%; dentistry was the most frequent field (61.4%), followed by general medicine (26.4%). Because the joint administration provided, for each respondent, both ATBEQ subscale scores and the percentage of “It depends” responses across the 35 scenarios, this dataset-and not the samples described above-was used for the K-means cluster analysis, linear discriminant analysis, and regression analysis. Because participation in all component studies was anonymous, the degree of individual overlap between the 244-participant cohort and the 277-participant sample cannot be determined; this is acknowledged as a limitation. Therefore, the n = 277 sample refers to the profile-based analyses [6], whereas the n = 244 cohort refers to the scenario-based and adapted DIT comparative analyses [4,5].

### 2.4. Conceptual Synthesis Procedure

The four source studies were treated as complementary empirical components rather than as datasets for statistical pooling. For each source study, the sample, instrument, principal findings relevant to ethical decision-making, and methodological limitations were considered. The findings were organized into four complementary evidence domains: (1) business-ethics orientations; (2) moral reasoning in structured ethical dilemmas; (3) scenario-based conditional ethical responding; and (4) multidimensional ethical reasoning profiles.

The synthesis then examined points of convergence and complementarity across these domains and interpreted them in relation to three educational dimensions relevant to entrepreneurial dental practice: individual cognitive-ethical orientation, entrepreneurial/practice-sustainability pressures, and normative-deontological constraints. These dimensions formed the conceptual basis of the three filters incorporated into the G.D.C. model.

The synthesis was conceptual and interpretive. No pooled effect estimates, meta-analysis, reanalysis of individual-level raw data, or new inferential statistical tests were performed. Consequently, the G.D.C. model should be understood as an empirically informed pedagogical framework generated from the integrated interpretation of previously published findings rather than as a statistically derived or externally validated predictive model.

### 2.5. Participants and Recruitment

Participants were recruited from academic and professional healthcare settings in Romania. In the 244-participant cohort, student participants were senior healthcare students, primarily from medicine and dentistry programs, and practitioner participants were licensed clinicians actively engaged in clinical practice [4,5]. In the 277-participant profile sample, participants were healthcare professionals and medical or dental students recruited online through professional networks, academic institutions, and social media channels [6].

Inclusion criteria were: (1) age of at least 18 years; (2) enrollment in a healthcare-related university program or active clinical practice as a healthcare professional; (3) ability to complete the questionnaire; and (4) provision of informed consent.

Exclusion criteria were: (1) refusal or absence of informed consent; (2) incomplete questionnaires preventing calculation of the main study variables; (3) duplicate responses; and (4) absence from the predefined target groups. For scale-based analyses, only cases with complete data for the relevant subscales were included. Missing data were handled through analysis-specific exclusion, meaning that incomplete cases were excluded only from the analysis for which the required variables were missing.

### 2.6. Instruments

#### 2.6.1. ATBEQ

Business-ethics attitudes were assessed using the Attitudes Toward Business Ethics Questionnaire (ATBEQ). The instrument includes 30 practical scenarios rated on a five-point Likert scale, from 1 = totally disagree to 5 = totally agree. The measured dimensions were Machiavellianism, moral objectivism, social Darwinism, ethical relativism, and legalism. The ATBEQ was originally developed to assess attitudes toward business ethics and has been used to examine ethical orientations such as Machiavellianism, objectivism, social Darwinism, ethical relativism, and legalism [7,8,9]. In the profile-based dataset, the instrument demonstrated good internal reliability (Cronbach’s α = 0.81) [6].

In the healthcare adaptation, respondents were instructed to analyze the scenarios from the perspective of their professional field. The ATBEQ was used to examine whether business-ethics philosophies originally developed in commercial contexts also structure ethical reasoning among healthcare students and practitioners. Throughout this study, the legalism dimension is referred to as Idealism/Legalism, following the labeling used in the profile-based source study, in which this subscale captured both rule-based and idealistic, harm-avoidant attitudes [6]; this term should not be confused with idealism as defined in ethical position theory [10].

#### 2.6.2. Adapted DIT-Based Moral Reasoning Assessment

Moral reasoning was assessed using a structured questionnaire adapted from the Defining Issues Test-2 (DIT-2), grounded in the neo-Kohlbergian tradition [11,12,13]. The instrument included three hypothetical but realistic moral dilemmas-the Doctor’s Dilemma, Jan and the Drug, and the Fugitive-each followed by 12 statements reflecting different moral reasoning patterns. The adaptation consisted of translation and back-translation by bilingual researchers, review by a panel of three experts in bioethics and medical education, and pilot testing with 15 participants [5]. Standard DIT-2 developmental indices (P-score, N2) were not computed; analyses were conducted at the level of individual decisions and argument ratings. The instrument is therefore referred to throughout as an adapted DIT-based assessment rather than a standard DIT-2 administration.

The Doctor’s Dilemma involved a terminally ill patient requesting an overdose of morphine to end suffering. Jan and the Drug involved access to life-saving medication when the pharmacist refuses to reduce the price. The Fugitive dilemma involved a convict who escapes, reintegrates into society, becomes successful, and contributes to the community.

For each dilemma, participants completed a decision task, rated the importance of each of the 12 arguments on a five-point scale, and ranked the four most important arguments [5]. The adapted DIT component was used to examine the relative weight given to justice, law, social order, autonomy, compassion, and professional responsibility.

#### 2.6.3. Scenario-Based Ethical Questionnaire

Scenario-based ethical decision-making was assessed using a 35-item questionnaire originally developed and validated in French settings by Vincent Richeux and Véronique Duquéroy, and adapted into Romanian through forward–backward translation, expert review, and pilot testing [4]. The instrument covers seven domains of clinical ethics: informed consent and transparency; confidentiality and mandatory reporting; medical error and professional responsibility; end-of-life care and allocation of limited resources; external influences and conflicts of interest; moral and cultural norms; and ethics in exceptional circumstances. Internal consistency in the Romanian samples was acceptable to good (Cronbach’s α = 0.71–0.84 across domains) [4].

Each item presented a hypothetical clinical scenario with three response options: Yes, No, and It depends. Conditional responses were retained as a separate analytical category. They were not recoded as missing or neutral answers. This allowed the analysis to distinguish categorical responses from conditional responding, without assuming that the latter represents a validated form of contextual ethical competence.

### 2.7. Variables and Outcome Measures

The main outcome in the scenario-based analysis was the type of ethical response selected by participants. Responses were coded as affirmative, negative, or conditional. The conditional category included “It depends” responses.

For the adapted DIT component, the main outcomes were differences between students and practitioners in the importance assigned to selected moral arguments and in dilemma-level response patterns [5].

For the ATBEQ component, the main variables were the five ethical philosophy subscale scores: Machiavellianism, moral objectivism, social Darwinism, ethical relativism, and legalism.

For the cluster analysis, standardized scores on the five ATBEQ subscales and the percentage of “It depends” responses were used to identify ethical reasoning profiles [6]. For the regression analysis, the dependent variable was the percentage of “It depends” responses across the ethical scenarios, and the predictors were the five ATBEQ subscale scores.

This variable was computed for each respondent by dividing the number of “It depends” responses by the total number of valid ethical scenario responses provided by that respondent and multiplying by 100, yielding a percentage between 0 and 100. The resulting percentage was used as a continuous outcome variable in the regression analysis. This approach allowed conditional ethical responding to be analyzed quantitatively while preserving the distinction between categorical responses and context-dependent responses.

### 2.8. Statistical Analysis

Data were analyzed in the source studies using IBM SPSS Statistics version 27 (scenario-based and profile-based components [4,6]) and MedCalc version 23.3.7 (adapted DIT component [5]). Descriptive statistics were used to summarize participant characteristics and questionnaire responses. Categorical variables were reported as frequencies and percentages. Continuous variables were reported as means and standard deviations or medians and interquartile ranges, depending on distribution.

Between-group comparisons of the three-category responses (Yes/No/It depends) were performed using Pearson’s chi-square test (df = 2), with Fisher’s exact test applied when expected cell counts were insufficient. Effect sizes (Cramér’s V) with 95% confidence intervals were reported, and exploratory logistic regression models adjusting for age, gender, and specialty yielded similar group patterns [4]. In the adapted DIT component, five-point argument-rating distributions were tested using chi-square tests with four degrees of freedom [5]. Statistical significance was set at *p* < 0.05. No correction for multiple comparisons was applied across the 35 scenario items or the DIT argument items; these comparisons are therefore interpreted as exploratory.

K-means cluster analysis was performed in the profile-based source study on six standardized variables: the five ATBEQ subscale scores and the percentage of “It depends” responses [6]. Standardization to z-scores was used to ensure equal scale contribution. The four-cluster solution (silhouette = 0.71) showed separation across the variables used to generate the clusters. Because these same variables were used in the clustering procedure, the observed between-cluster differences should be interpreted as descriptive characteristics of the solution rather than as independent evidence of cluster validity.

Linear discriminant analysis was used as an internal classification check for the K-means-derived clusters [6]. No external, split-sample, or bootstrap validation of the cluster solution was performed; therefore, the identified profiles are treated as exploratory descriptive patterns. Because the discriminant functions were estimated on the same data from which the clusters were derived, classification accuracy should be interpreted as an internal consistency indicator rather than as independent validation.

Multiple linear regression was used in the profile-based source study to predict the percentage of “It depends” responses from the five ATBEQ subscale scores [6]. Variance inflation factors were calculated to assess multicollinearity. Regression coefficients, standard errors, standardized beta coefficients, 95% confidence intervals, *p*-values, R^2^, and adjusted R^2^ were reported.

### 2.9. Development of the G.D.C. Model

The G.D.C. framework was developed by conceptually mapping findings from the four source studies and relevant literature on bioethics, moral development, ethical position theory [10,14], and business ethics into three domains: cognitive-ethical orientation, entrepreneurial/practice-sustainability pressures, and normative-deontological constraints. These domains formed the three interacting filters of the framework. The structure was conceptually derived rather than statistically generated and remains exploratory and hypothesis-generating.

The three-filter structure was retained because the conceptual mapping converged on three higher-order domains: individual cognitive-ethical orientation, entrepreneurial/practice-sustainability pressures, and normative-deontological constraints. All three domains were informed by the source findings, while their organization into a dentistry-oriented framework and the shared decision-making and reassessment process were additionally informed by ethical literature and author interpretation. The framework was not statistically derived.

The cognitive-ethical filter refers to individual moral orientation, ethical sensitivity, moral judgment, and professional values. The entrepreneurial filter includes treatment costs, clinic sustainability, technology investment, patient affordability, time pressure, and market competition. The normative-deontological filter includes legal obligations, professional codes, informed consent, institutional procedures, data protection, and standards of care.

### 2.10. Ethical Approval

The study was conducted in accordance with the Declaration of Helsinki and was approved by the Ethics Committee of the “Victor Babeș” University of Medicine and Pharmacy, Timișoara, Romania (protocol code No. 87/20.10.2023 rev. 2025; approval date: 20 October 2023). All participants provided informed consent before completing the questionnaire. Participation was voluntary and anonymous.

The present study is a conceptual synthesis of previously published studies and involved no new participant recruitment or data collection. Ethical approval for the original studies is reported in the respective source publications [3,4,5,6]. The current conceptual synthesis involved no new analysis of identifiable participant data and therefore did not require additional participant consent. Because participation in the source studies was anonymous, the degree of individual overlap between the 244-participant cohort and the 277-participant sample cannot be determined; consequently, these samples should not be regarded as fully independent bodies of evidence.

## 3. Results

### 3.1. Empirical Sources Informing the Conceptual Framework

The development of the conceptual framework was informed by four previously published empirical components ATBEQ-based business-ethics attitudes [3], adapted DIT-based moral reasoning [5], scenario-based ethical decision-making [4], and profile-based analyses of a jointly administered ATBEQ–scenario dataset [6] (Table 1). The scenario-based and adapted DIT comparative analyses refer to the 244-participant cohort, whereas the profile-based analyses used the separate 277-participant sample described in Section 2.3.

This structure allowed the study to examine ethical reasoning from four complementary perspectives: general business-ethics orientation, moral reasoning in classical ethical dilemmas, practical responses to clinical vignettes, and multidimensional ethical reasoning profiles. The empirical components informing the present conceptual framework are summarized in Table 1.

### 3.2. Participant Characteristics in the Scenario-Based and Adapted DIT Components

The scenario-based and adapted DIT analyses included 244 participants: 51 senior students and 193 practicing clinicians (242 valid cases were retained in the adapted DIT component after data cleaning) [4,5]. Among students, 42 were enrolled in dentistry and 9 in general medicine. Among practitioners, 132 were dentists and 61 were general practitioners. Overall, 174 participants were from dentistry and 70 from general medicine.

The sample was predominantly female. Among students, 43 were female and 8 were male. Among practitioners, 147 were female and 46 were male. Professional experience among practitioners ranged from 1 to 28 years, with a median of 12 years. The demographic and professional characteristics of this sample are presented in Table 2.

### 3.3. Scenario-Based Ethical Reasoning

The quantitative findings presented in this subsection were previously reported in the scenario-based study [4] and are reproduced here without recalculation. Their inclusion in the present study serves to inform the conceptual development of the G.D.C. framework rather than to present new empirical analyses.

Across the 35 scenario-based ethical items, conditional responding was highly frequent: “It depends” represented 47.8% of pooled responses, making it the most common response category, followed by “Yes” (33.5%) and “No” (18.7%) [4]. Whether this response pattern reflects contextual considerations, uncertainty, avoidance, or indecision cannot be determined from the quantitative data alone. It is therefore described as conditional responding and should not be interpreted as evidence of contextual ethical competence.

Several statistically significant differences were identified between students and practitioners (Table 3); given that 35 uncorrected comparisons were performed, these results should be regarded as exploratory. Students were more likely than practitioners to support mandatory annual influenza vaccination for healthcare workers (52.94% vs. 32.64%; χ^2^(2) = 7.16, *p* = 0.028, Cramér’s V = 0.17). Students were also more likely to support authorization of a regulated organ market (76.47% vs. 62.18%; χ^2^(2) = 6.32, *p* = 0.043, V = 0.16). Practitioners were more likely to support higher insurance contributions for individuals with risky health behaviors (48.70% vs. 23.53%; χ^2^(2) = 11.63, *p* = 0.003, V = 0.22). These differences remained robust in exploratory logistic regression models adjusting for age, gender, and specialty [4]. Three of the 35 uncorrected scenario comparisons reached nominal statistical significance. Given the number of comparisons and the absence of multiplicity correction, these findings should be considered exploratory and hypothesis-generating rather than evidence of stable differences in ethical orientation between students and practitioners.

Given the absence of multiplicity correction, these nominal between-group differences are treated as exploratory and hypothesis-generating and should not be interpreted as evidence of stable differences in ethical orientation between students and practitioners.

### 3.4. Adapted DIT-Based Moral Reasoning

The findings presented in this subsection were previously reported in the adapted DIT study [5]. The adapted DIT findings showed differences between students and practitioners in moral reasoning about law, justice, punishment, and autonomy [5]. In the Fugitive dilemma, practitioners prioritized law- and punishment-oriented arguments more strongly than students. Significant differences were identified for items reflecting the view that past crimes cannot be erased by later good deeds, that punishment must be applied consistently regardless of reintegration, and that a person must pay for a crime even if later becoming a good citizen.

In the Doctor’s Dilemma, practitioners gave significantly higher scores to autonomy-related arguments in end-of-life decision-making (χ^2^ = 9.76, df = 4, *p* = 0.045) [5]. This finding indicates that practitioner reasoning was not uniformly more rigid or conservative. Practitioners appeared more legalistic in justice-related scenarios, but also more sensitive to patient autonomy in clinically complex end-of-life contexts.

Overall, these exploratory differences may reflect domain-specific variation in ethical reasoning. Because the data are cross-sectional, they cannot establish that professional experience caused the observed differences.

### 3.5. ATBEQ-Based Business-Ethics Attitudes

The findings presented in this subsection were previously reported in the ATBEQ study [3]. The ATBEQ component showed that moral objectivism was the dominant business-ethics orientation among Romanian medical students and doctors. This suggests that healthcare participants tended to favor rational action grounded in objective moral standards. In this context, moral objectivism may reflect a professional preference for consistency, impartiality, patient welfare, and adherence to standards.

Legalism ranked differently across groups and appeared particularly relevant for understanding professional experience. Prior findings indicated a significant difference in legalism between medical students and doctors, while other ATBEQ dimensions were more similar across the two healthcare groups. This exploratory difference may be associated with professional, cohort, training, or workplace characteristics; however, the cross-sectional data do not establish an effect of clinical exposure.

The ATBEQ findings also support the educational relevance of business ethics in healthcare. Attitudes toward Machiavellianism, moral objectivism, legalism, ethical relativism, and social Darwinism may influence how future dentists understand private-practice sustainability, patient communication, professional responsibility, and commercial pressures.

### 3.6. Ethical Reasoning Profiles

The findings presented in this subsection were previously reported in the profile-based study [6]. The ethical reasoning profiles were derived in the source study through K-means cluster analysis of six standardized variables-the five ATBEQ subscale scores and the percentage of “It depends” responses-in the 277-participant sample [6]. The four-cluster solution (silhouette = 0.71) showed separation across the variables used to generate the clusters. Because these same variables were used in the clustering procedure, the observed between-cluster differences should be interpreted as descriptive characteristics of the solution rather than as independent evidence of cluster validity.

For the present study, neutral educational labels were assigned to the four profiles identified in the source study [6]: Strategic Idealists (labeled High Machiavellian Idealists in the source study), Contextual Pragmatists (Pragmatic Relativists), Evidence-Oriented Objectivists (Context-Sensitive Objectivists), and Low-Integration Idealists (Ethical Purists). The four profiles are summarized in Table 4.

Linear discriminant analysis was performed in the source study as an internal descriptive check of cluster separation. Because the discriminant functions were derived from the same dataset and variables used to generate the clusters, the resulting classification accuracy does not constitute independent validation. The four profiles should therefore be regarded as provisional descriptive patterns requiring external validation and stability testing. The first function separated clusters by overall moral-philosophical orientation, the second differentiated participants primarily by conditional responding, and the third contrasted ethical relativism with Idealism/Legalism [6].

### 3.7. Regression Analysis of Conditional Ethical Responding

In the source study, multiple linear regression was conducted with the percentage of “It depends” responses as the dependent variable and the five ATBEQ subscale scores as predictors [6]. The overall model was not statistically significant (*p* = 0.08) and its explanatory power was low (R^2^ = 0.04; adjusted R^2^ = 0.02). Variance inflation factors ranged from 1.21 to 1.46, indicating no multicollinearity.

Idealism/Legalism was the only individually significant predictor of conditional responding (B = −0.69 percentage points per scale point, SE = 0.30, 95% CI −1.28 to −0.11, β = −0.15, *p* = 0.021). No other ATBEQ subscale reached statistical significance. Because the overall model was not significant, this coefficient should be interpreted as an exploratory finding requiring replication. The regression results are summarized in Table 5.

Although Idealism/Legalism was individually associated with fewer conditional responses, this finding should be interpreted cautiously because the overall regression model was not statistically significant (R^2^ = 0.04; adjusted R^2^ = 0.02). The association is therefore considered exploratory and requires replication. This supports the need for a multi-filter model in which ethical decisions are shaped by individual moral orientation, clinical context, economic pressure, institutional norms, and legal constraints.

### 3.8. Empirical Basis for the G.D.C. Model

The integrated findings informed the development of the G.D.C. model as an exploratory educational framework; they should not be interpreted as statistically validating the model. Three findings are central.

**Figure 1:**
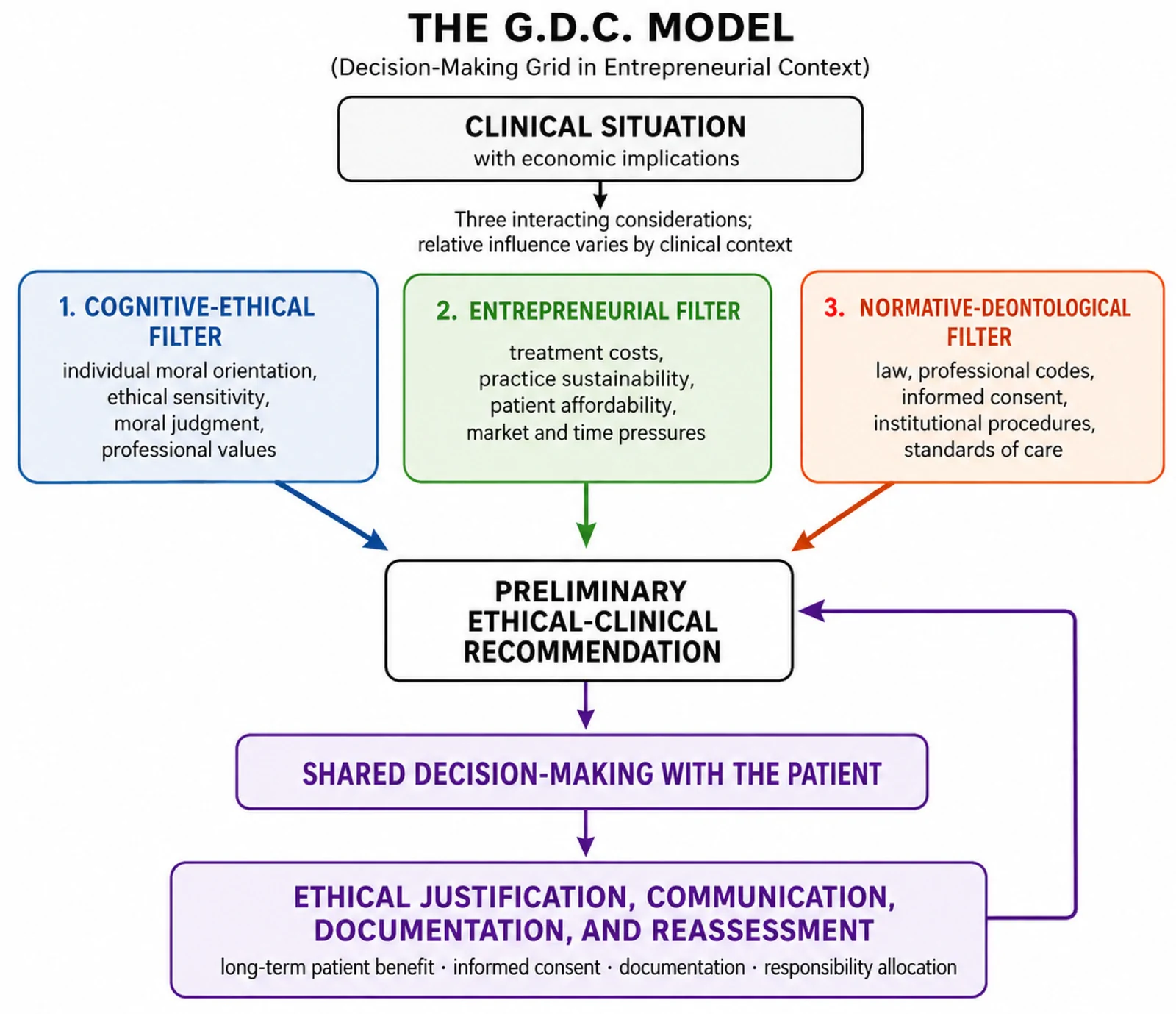
The G.D.C. model (Grid for Decision-making in entrepreneurial Contexts). The G.D.C. framework for ethical decision-making in entrepreneurial dentistry. The framework integrates three interacting considerations-cognitive-ethical, entrepreneurial, and normative-deontological-whose relative influence may vary according to the clinical context. These considerations inform a preliminary ethical-clinical recommendation, followed by shared decision-making with the patient and an iterative process of ethical justification, communication, documentation, and reassessment.

The proposed G.D.C. model is presented in Figure 1. The figure illustrates how a clinical situation with economic implications is processed through three interacting filters: the cognitive-ethical filter, the entrepreneurial filter, and the normative-deontological filter. The three filters represent interacting considerations whose relative importance may vary according to the clinical context. Their integration informs a preliminary recommendation that is subsequently discussed with the patient through shared decision-making. Ethical justification, communication, documentation, and reassessment are incorporated as part of an iterative process, allowing the recommendation to be revised when new clinical information, patient preferences, or contextual factors emerge.

First, exploratory differences between students and practitioners were observed across selected ethical domains, but these differences cannot be attributed causally to professional exposure. Second, conditional responding was frequent, although its meaning cannot be determined from the quantitative data alone. Third, the cluster analysis yielded provisional descriptive patterns that require external validation. Together, these observations informed the conceptual development of the G.D.C. framework but do not validate it empirically.

Taken together, these findings provided the conceptual basis for the central premise of the G.D.C. model: ethical decision-making in entrepreneurial dentistry is shaped by the interaction of individual ethical orientation, entrepreneurial contextual pressure, and normative-deontological regulation. However, because the overall regression model was not statistically significant and had limited explanatory power, the model should not be presented as validated in a predictive sense. It should be described as a conceptual and pedagogical framework requiring further empirical testing.

## 4. Discussion

This study synthesized empirical findings on business-ethics attitudes, moral reasoning, scenario-based ethical decision-making, and ethical reasoning profiles among Romanian healthcare students and practitioners [3,4,5,6], with a specific interpretive focus on dental education and entrepreneurial dental practice. The findings support the working hypothesis that ethical decision-making in entrepreneurial dentistry cannot be explained adequately by a purely principlist model or by a purely economic model. Rather, it appears to operate as a hybrid process in which professional values, legal constraints, clinical context, patient-related factors, and private-practice realities interact.

The frequent use of “It depends” is particularly relevant. In dental practice, many ethical questions cannot be answered responsibly without considering diagnosis, prognosis, affordability, patient autonomy, informed consent, legal requirements, available treatment alternatives, and long-term maintenance. Conditional responding may reflect consideration of contextual factors, but it should not be interpreted as a validated measure of contextual ethical competence. It may also reflect uncertainty, avoidance, or insufficiently structured ethical reasoning. It may also indicate uncertainty, avoidance, or insufficiently structured ethical reasoning. This distinction has direct implications for dental education, because students must be trained not only to recognize complexity, but also to justify conditional decisions using explicit ethical, clinical, and legal criteria.

The observed differences between students and practitioners should not be interpreted simplistically as ethical erosion [15,16,17]. The observed exploratory differences may suggest that students were more likely to support some collective or principle-driven positions, while practitioners appeared more attentive to legal, institutional, and resource-related considerations. The observed exploratory differences may be consistent with pragmatic recalibration rather than ethical decline; however, this interpretation remains hypothetical. Because the data are cross-sectional, the observed differences cannot be attributed directly to professional experience. Age, cohort, training, selection, and workplace factors may also contribute to these patterns.

Professional experience exposes clinicians to legal liability, resource limitations, patient non-adherence, institutional expectations, practice sustainability, and the real consequences of treatment decisions. These pressures may alter how ethical principles are prioritized without eliminating moral concern. This interpretation is consistent with the findings of the adapted DIT-based and scenario-based studies, in which practitioners showed stronger law-based reasoning in some dilemmas, but also greater sensitivity to autonomy-related arguments in clinically complex contexts [4,5].

For dental education, this finding is important. Ethics curricula should not aim only to preserve student idealism. They should also prepare students to apply ethical principles under conditions of uncertainty, legal constraint, economic pressure, and clinical ambiguity. The educational task is not to remove complexity, but to help students reason within it without compromising patient welfare.

The ATBEQ findings are important because dentists often operate simultaneously as clinicians and private-practice actors. Moral objectivism as a dominant orientation suggests that medical students and doctors tend to value rational, standard-based ethical reasoning [3]. In a healthcare context, this may support professional consistency, impartiality, and patient welfare.

At the same time, the presence of Machiavellianism, relativism, legalism, and social Darwinism as measurable dimensions shows that business-ethics attitudes cannot be ignored in healthcare and dental education. This caution is particularly important because Machiavellianism and related organizational risk factors have been associated with unethical decision-making in professional contexts [18,19,20,21,22]. Private dentistry requires communication, marketing, treatment acceptance, financial planning, and organizational sustainability. These activities are not unethical in themselves. They become ethically problematic when they distort informed consent, exaggerate benefits, minimize risks, or prioritize revenue over patient welfare.

The Strategic Idealists profile illustrates this tension. Strategic communication can support patient understanding and adherence to clinically indicated treatment. However, the present interpretation does not frame Machiavellianism as ethically desirable. Rather, it recognizes that strategic orientation may coexist statistically with idealistic attitudes, while emphasizing that any persuasive communication in dentistry must remain transparent, evidence-based, non-coercive, and compatible with informed consent. Dental education should therefore explicitly teach the boundary between ethical recommendation and commercial persuasion, a distinction long emphasized in chairside approaches to dental ethics [23]. 

Recent dental literature further emphasizes the importance of contextual and patient-centered ethical decision-making. Case-based approaches have been used to examine moral reasoning in dental students, while recent studies have highlighted professional identity formation as an important component of dental education. Research in private dental practice has also identified overtreatment and changing boundaries of treatment necessity as relevant ethical concerns, reinforcing the need to address commercial pressures alongside patient welfare and shared decision-making [2,24,25,26].

Idealism/Legalism showed an isolated exploratory association with fewer conditional responses. However, because the overall regression model was not statistically significant and explained only 4% of the variance, this finding should not be interpreted as evidence of a robust predictive relationship. In dental practice, legal and deontological norms are essential. They define non-negotiable boundaries related to informed consent, confidentiality, documentation, treatment standards, patient safety, and professional accountability.

However, legalism cannot replace ethical reasoning. The regression model explained only 4% of the variance in conditional responding and was not statistically significant overall. This low explanatory power is important. It indicates that ethical decision-making cannot be reduced to one attitudinal dimension. This interpretation is consistent with broader moral psychology models emphasizing intuitive, reflective, domain-specific, and process-based components of moral judgment [27,28,29,30,31]. A legally compliant decision may still be poorly communicated, insufficiently patient-centered, or commercially biased. Conversely, contextual flexibility may be valuable, but ethically unsafe if it lacks clear professional boundaries. Dental education should therefore teach students to combine legal clarity with contextual ethical judgment.

The educational implications associated with the ethical profiles should be considered exploratory hypotheses rather than established profile-specific needs. Prospective studies are required to determine whether such tailored educational approaches are effective.

The four ethical reasoning profiles represent provisional descriptive patterns rather than validated learner types. Their potential educational implications should therefore be considered exploratory hypotheses. For example, the observed profiles may help generate hypotheses regarding training needs related to ethical persuasion, deontological reasoning, shared decision-making, socioeconomic sensitivity, and structured ethical decision-making. These profile-specific educational approaches require prospective validation before being used to guide individualized instruction.

These findings support a case-based approach to dental ethics education. Prior research on professional ethics education and organizational ethics training also supports applied, context-sensitive, and values-based approaches rather than purely abstract instruction [32,33,34]. Teaching should include realistic treatment-planning dilemmas in which several options are clinically acceptable but differ in cost, esthetic outcome, prognosis, long-term maintenance, and patient burden. Students should be required to justify recommendations, present reasonable alternatives, disclose uncertainty, and identify potential conflicts of interest.

The COVID-19 pandemic accelerated the use of online and blended learning in dental education. These approaches, including Blended Intensive Programmes (BIPs), remain relevant for ethics education by supporting international collaboration, case-based discussion, and exposure to diverse clinical and ethical perspectives [35,36,37].

A useful educational strategy would be to ask students to analyze each case through four questions: (1) What is clinically indicated? (2) What does the patient understand, value, and afford? (3) What economic or institutional pressures may influence the recommendation? (4) What legal and professional standards define the non-negotiable limits of the decision? This structure would make the G.D.C. model operational for classroom, simulation, and clinical ethics training.

The G.D.C. framework could be integrated across dental education with increasing complexity. At the undergraduate level, students could apply the three filters to treatment-planning cases addressing clinical, ethical, economic, and legal considerations. Simulation and OSCE activities could further develop communication and justification skills. At the postgraduate level, the framework could be used for more complex, specialty-specific cases through case discussions, reflective exercises, and review of anonymized clinical situations. The ethical profiles should be used only as exploratory educational tools, not as fixed learner categories.

The G.D.C. model integrates the empirical findings into a three-filter framework. The cognitive-ethical filter includes moral orientation, ethical sensitivity, moral judgment, and professional values. The entrepreneurial filter includes cost, sustainability, market competition, technological investment, treatment acceptance, and patient affordability. The normative-deontological filter includes law, professional codes, informed consent, institutional procedures, data protection, and standards of care.

The framework incorporates an iterative process in which the three considerations inform a preliminary ethical-clinical recommendation. This recommendation is then discussed with the patient through shared decision-making, followed by ethical justification, communication, documentation, and reassessment. The recommendation may be revised when patient preferences, clinical information, or contextual factors change.

The G.D.C. model is therefore useful primarily as a pedagogical tool. Its purpose is not to predict behavior with precision. Its purpose is to help students and practitioners identify the forces shaping ethically complex treatment decisions. In this sense, the model translates empirical findings into a structured educational framework for teaching ethics in private dental practice.

A strength of this study is its integrative perspective. By combining findings from ATBEQ-based business-ethics assessment, adapted DIT-based moral reasoning, scenario-based ethical decision-making, and profile-based cluster analyses [3,4,5,6], it provides a broader interpretation of ethical reasoning than any single instrument could offer. The study also addresses a relevant educational problem: how to prepare dental students for ethical decision-making in entrepreneurial practice contexts.

However, several limitations must be acknowledged. First, this study is an exploratory conceptual framework paper based on previously published empirical findings; it reports no new primary data or statistical analyses.; it reports no new primary data, and all quantitative results are drawn from the four published source studies [3,4,5,6]. Second, the empirical sources used related but not identical samples, and because data collection was anonymous, the degree of participant overlap between the 244-participant cohort and the 277-participant profile sample cannot be determined. Third, the scenario-based comparisons involved 35 uncorrected tests, of which only three reached nominal significance; these findings are exploratory. Fourth, the adapted DIT assessment did not compute standard developmental indices (P-score, N2), limiting comparability with the wider DIT literature. Fifth, the cluster solution is exploratory and should not be interpreted as identifying fixed personality types, and the overall regression model predicting conditional responding was not statistically significant. Sixth, “It depends” responses were analyzed quantitatively, but their qualitative meaning remains uncertain. Finally, the findings come from Romanian healthcare education and practice and may not generalize directly to other institutional, cultural, or healthcare-market contexts. The G.D.C. framework should not be interpreted as having been empirically validated specifically in entrepreneurial dentistry. It represents a conceptual extrapolation from findings obtained in mixed Romanian healthcare samples, including medical and dental students and practitioners. Its applicability to private dental practice therefore requires prospective, dental-specific validation.

Another limitation is that the G.D.C. model has not yet been prospectively tested as an educational intervention. Its value is currently conceptual and pedagogical. Future studies should examine whether using this model in dental ethics education improves students’ ability to identify conflicts of interest, justify treatment recommendations, communicate alternatives, and balance patient welfare with private-practice sustainability.

The applicability of the G.D.C. framework may also vary across healthcare systems and cultural settings. The relative importance of its three filters is unlikely to be identical in predominantly private-pay, insurance-based, or publicly funded dental systems. In private-pay environments, patient affordability, treatment acceptance, market competition, and commercial incentives may be particularly salient, whereas insurance-based systems may place greater emphasis on reimbursement rules, coverage restrictions, and insurer-related constraints. In predominantly public systems, resource allocation, waiting times, institutional budgets, and organizational policies may represent more prominent contextual pressures. 

Cultural differences may likewise influence expectations regarding patient autonomy, family involvement, professional authority, disclosure, and the perceived legitimacy of commercial considerations in healthcare. Accordingly, the three-filter structure of the G.D.C. model may provide a transferable heuristic, but the content and relative weighting of its components should be adapted to local regulatory, economic, professional, and cultural conditions rather than assumed to be universal.

Future research should test the G.D.C. model prospectively in dental students, residents, early-career dentists, experienced practitioners, and clinic owners. Longitudinal designs would help determine whether ethical reasoning profiles change during clinical training and professional practice.

Mixed-methods studies are also needed. Interviews, written justifications, think-aloud protocols, and simulated treatment-planning encounters could clarify whether “It depends” responses reflect principled consideration of contextual factors, uncertainty, avoidance, or ethical indecision. Such methods would provide stronger evidence than quantitative response categories alone.

Future studies should also examine whether G.D.C.-based ethics training improves measurable educational outcomes, such as ethical sensitivity, quality of treatment-plan justification, transparency in patient communication, and recognition of commercial influence. Cross-cultural research is needed to assess whether the model is applicable beyond Romanian healthcare education and private-practice contexts. This is especially relevant because moral reasoning and ethical judgments may vary across cultural and institutional contexts [38].

## 5. Conclusions

Ethical decision-making in entrepreneurial dentistry may involve the interaction of moral reasoning, patient context, legal obligations, and practice-related pressures. The previously published findings considered in this study suggest differences between students and practitioners, but these observations remain exploratory and should not be interpreted as evidence of causal effects of professional experience.

Conditional responding (“It depends”) was frequent, but its meaning remains uncertain and may reflect contextual considerations, uncertainty, avoidance, or indecision. Likewise, the observed association between Idealism/Legalism and fewer conditional responses arose within a non-significant regression model with low explanatory power and therefore requires replication.

The G.D.C. framework is a theory-informed, hypothesis-generating educational framework derived from mixed Romanian healthcare samples. It has not been validated as a predictive model or specifically in entrepreneurial dentistry. Prospective, dental-specific, cross-cultural, and educational studies are needed to evaluate its applicability and usefulness in dental ethics education.

## Figures and Tables

**Table 1:** Empirical components integrated in the present study, including the source dataset, sample size, instruments, previously published findings, and their role in the development of the G.D.C. framework.

Empirical Component	Source Study	Sample	Instrument/Variables	Previously Published Analyses/Findings	Role in the Current Study/New Contribution
Business-ethics attitudes	ATBEQ study [3]	n = 353 (53 medical students, 192 doctors, 108 management students)	ATBEQ; Machiavellianism, moral objectivism, social Darwinism, ethical relativism, legalism	Group patterns in business-ethics orientations were previously analyzed and reported in [3].	Findings are conceptually reinterpreted in relation to ethical and entrepreneurial influences relevant to dental practice and incorporated into the G.D.C. framework.
Moral reasoning	Adapted DIT study [5]	n = 244 (242 valid cases)	Adapted DIT; 3 dilemmas × 12 arguments	Student–practitioner differences in moral reasoning and argument prioritization were previously reported in [5].	Findings are used to inform the cognitive-ethical and normative-deontological dimensions of the G.D.C. framework.
Scenario-based ethics	Vignette-based study [4]	n = 244	35-item ethical scenario questionnaire; Yes/No/“It depends” responses	Scenario-response distributions and student–practitioner comparisons were previously reported in [4].	Conditional responding is conceptually reconsidered as a potential marker of context-sensitive decision-making, while remaining distinct from validated ethical competence.
Ethical reasoning profiles	Joint ATBEQ + scenario study [6]	n = 277	ATBEQ subscales and percentage of “It depends” responses	Cluster profiles and regression analyses were previously reported in [6].	Previously identified patterns are reinterpreted for educational purposes and used to generate hypotheses for case-based dental ethics training.

Note: All quantitative findings and statistical analyses summarized in this table were previously reported in the cited source studies [3,4,5,6]. No new statistical analyses were conducted for the present study. The original contribution of the current work is the conceptual reinterpretation and organization of these findings within the G.D.C. framework for dental ethics education.

**Table 2:** Sociodemographic and professional characteristics of the 244-participant cohort (scenario-based and adapted DIT components).

Variable	Students (n = 51)	Practitioners (n = 193)	Total (n = 244)
Male, n (%)	8 (15.69)	46 (23.83)	54 (22.13)
Female, n (%)	43 (84.31)	147 (76.17)	190 (77.87)
Dentistry, n (%)	42 (82.35)	132 (68.39)	174 (71.31)
General medicine, n (%)	9 (17.65)	61 (31.61)	70 (28.69)
Median years of experience (IQR)	-	12 (6–21)	-

**Table 3:** Nominally significant scenario-based differences between students and practitioners.

Topic/Scenario Topic	Scenario/Item	Students (%)	Practitioners (%)	χ^2^ (df = 2), *p*, Cramér’s V
Public health	Support for mandatory annual influenza vaccination for healthcare workers	52.94	32.64	7.16; *p* = 0.028; V = 0.17
Economic justice	Support for higher insurance contributions for individuals with risky health behaviors	23.53	48.70	11.63; *p* = 0.003; V = 0.22
Transplantation policy	Support for authorizing a regulated organ market	76.47	62.18	6.32; *p* = 0.043; V = 0.16

Note: Complete Yes/No/“It depends” response distributions for all scenarios are reported in the original source study [4]. The present table summarizes only the exploratory comparisons relevant to the current conceptual framework.

**Table 4:** Ethical reasoning profiles identified through cluster analysis.

Profile	n	Main Characteristics	Exploratory Educational Hypothesis
Strategic Idealists	52	High Machiavellianism and high Idealism/Legalism; moderate-to-high moral objectivism; moderate conditional responding.	May benefit from training on the ethical limits of persuasion, transparent treatment communication, and informed consent.
Contextual Pragmatists	72	Moderate-to-high Machiavellianism, high ethical relativism, low Idealism/Legalism, highest “It depends” rate.	May require stronger deontological anchors and explicit boundaries between contextual flexibility and ethical compromise.
Evidence-Oriented Objectivists	69	Moderate ethical-orientation scores, moderate-to-high moral objectivism, low ethical relativism, lowest conditional responding.	May align with evidence-based decision-making, but should also be trained in patient vulnerability and socioeconomic sensitivity.
Low-Integration Idealists	84	Low scores across ethical dimensions and moderate-to-high conditional responding.	May benefit from structured case-based ethics training and stepwise ethical decision-making frameworks.

**Table 5:** Multiple regression predicting “It depends” responses.

Predictor/Model Parameter	B	SE	β	*p*-Value	Interpretation
Idealism/Legalism	−0.69	0.30	−0.15	0.021	Higher Idealism/Legalism was associated with fewer conditional responses (95% CI −1.28 to −0.11).
Other ATBEQ subscales	-	-	-	>0.10	No other ATBEQ subscale reached statistical significance.
Model fit	-	-	-	0.08	F(5,271) = 1.99, R^2^ = 0.04, adjusted R^2^ = 0.02; overall model not statistically significant.

## Data Availability

The data supporting the findings synthesized in this study are reported in the cited source studies [3,4,5,6]; the underlying datasets are available on request from the corresponding authors and are not publicly available due to privacy and ethical restrictions.

## Abbreviations

The following abbreviations are used in this study:

ATBEQ	Attitudes Toward Business Ethics Questionnaire
DIT-2	Defining Issues Test-2
G.D.C.	Grid for Decision-making in entrepreneurial Contexts
